# Mortality from suicide and other external cause injuries in China: a prospective cohort study

**DOI:** 10.1186/1471-2458-11-56

**Published:** 2011-01-27

**Authors:** Casey M Rebholz, Dongfeng Gu, Wenjie Yang, Jing Chen, Xigui Wu, Jian-feng Huang, Ji-chun Chen, Chung-Shiuan Chen, Tanika N Kelly, Xiufang Duan, Lydia A Bazzano, Jiang He

**Affiliations:** 1 Department of Epidemiology, Tulane University School of Public Health and Tropical Medicine, 1440 Canal Street, Suite 2000, New Orleans, LA 70112, USA; 2 Department of Medicine, Tulane University School of Medicine, 1430 Tulane Avenue, SL-12, New Orleans, LA 70112, USA; 3 Department of Evidence Based Medicine, Cardiovascular Institute and Fu Wai Hospital, Chinese Academy of Medical Sciences and Peking Union Medical College, and National Center for Cardiovascular Disease, 167 Beilishi Road, Beijing 100037, PR China

## Abstract

**Background:**

Premature death from suicide is a leading cause of death worldwide. However, the pattern and risk factors for suicide and other external cause injuries are not well understood. This study investigates mortality from suicide and other injuries and associated risk factors in China.

**Methods:**

A prospective cohort study of 169,871 Chinese adults aged 40 years and older was conducted. Mortality due to suicide or other external cause injuries was recorded.

**Results:**

Mortality from all external causes was 58.7/100,000 (72.3 in men and 44.4 in women): 14.1/100,000 (14.2 in men and 14.2 in women) for suicide and 44.6/100,000 (58.1 in men and 30.2 in women) for other external cause injuries. Transport accidents (17.2/100,000 overall, 23.4 in men and 10.8 in women), accidental poisoning (7.5/100,000 overall, 10.2 in men and 4.8 in women), and accidental falls (5.7/100,000 overall, 6.5 in men and 5.0 in women) were the three leading causes of death from other external cause injuries in China. In the multivariable analysis, male sex (relative risk [RR] 1.56, 95% confidence interval [CI] 1.03-2.38), age 70 years and older (2.27, 1.29-3.98), living in north China (1.68, 1.20-2.36) and rural residence (2.82, 1.76-4.51) were associated with increased mortality from suicide. Male sex (RR 2.50, 95% CI 1.95-3.20), age 60-69 years (1.93, 1.45-2.58) and 70 years and older (3.58, 2.58-4.97), rural residence (2.29, 1.77-2.96), and having no education (1.56, 1.00-2.43) were associated with increased mortality from other external cause injuries, while overweight (0.60, 0.43-0.83) was associated with decreased risk of mortality from other external cause injuries.

**Conclusions:**

External cause mortality has become a major public health problem in China. Developing an integrated national program for the prevention of mortality due to external cause injuries in China is warranted.

## Background

Premature death from suicide is among the leading causes of death in the world[1]. Given the large population size, China constitutes about one fifth of all suicides[1]. Phillips and colleagues reported a suicide mortality rate of 23 per 100,000 or 287,000 deaths due to suicide annually in China for 1995-1999[2]. This estimate is conservative compared to the suicide mortality rates of 30.3 and 31.0 per 100,000 for China in 1990 and 2000 respectively, as estimated by the Global Burden of Disease study[3]. In most countries, mortality rates due to suicide are higher among men than women[4]. However, this relationship is reversed in China, with suicide fatalities being more common among women than men[2,5-7]. In addition, some studies have demonstrated greater mortality due to suicide in rural areas than urban areas in China[2,5-8]. This unique pattern of suicide in China is attracting attention and requires further investigation.

The sociocultural understanding of suicide in China differs from that in the US. In China, attitudes towards suicide vary from tolerance to condemnation based on sociodemographic characteristics[9,10]. Some investigators found that approval of suicide is inversely associated with satisfaction of economic status[11,12]. Suicide is perhaps partly socially-sanctioned in China as a means to deal with social, economic, political, or moral adversity, particularly for Chinese women[6,10]. The sociocultural understanding and risk factors for suicide in China relate more to psychosocial stressors and interpersonal conflict than mental illness[13].

Injuries also constitute a significant public health problem. Approximately 5.8 million people worldwide die each year and even more people become disabled as a result of injuries[1]. Accidents are estimated to be the sixth leading cause of death after heart disease, cancer, cerebrovascular disease, pneumonia and influenza, and infectious diseases in China[14]. The leading causes of fatal unintentional injuries both worldwide and in China specifically are road traffic accidents, drowning, and falls[1].

Several studies have reported mortality from suicide and other external injuries in China based on regional data or government reports[1,2,5,15]. We conducted a large, prospective cohort study involving a representative sample of the general adult population 40 years of age and older to examine mortality and risk factors for suicide and other external cause injuries in China. A better understanding of the magnitude of mortality from suicide and other external injuries will provide scientific basis for effective prevention efforts in China.

## Methods

### Study population

In the 1991 China National Hypertension Survey, a multistage random cluster sampling design was used to select a representative sample of the general Chinese population aged 15 years and older from all 30 provinces in mainland China[16]. In 1999-2000, investigators from each province were invited to participate in the China National Hypertension Survey Epidemiology Follow-up study. Of the 30 provinces, 13 were not included in the follow-up study because study participants' contact information was not available. However, the sampling process was conducted independently within each province in the 1991 China National Hypertension Survey and the 17 provinces that were included in the follow-up study were evenly distributed in different geographic regions of China representing various stages of economic development. From the 17 provinces, 169,871 study participants (83,533 men and 86,338 women) aged 40 years or older at their baseline examination were eligible for the follow-up study. The baseline characteristics of study participants from the 17 provinces eligible for the follow-up study were similar to those in the 13 excluded provinces. From this population, a total of 158,666 (93.4%) study participants (or their proxies) were identified and interviewed in the follow-up study. Participants included in the final analysis were not different from the eligible study population regarding their baseline characteristics.

### Baseline examination

In 1991, baseline data were collected during a single clinic visit by specially trained physicians and nurses using standardized methods with a stringent level of quality control. Data on demographic characteristics, medical history, and lifestyle risk factors were obtained using a standardized questionnaire administered by trained staff. Employment status was categorized as unemployed, labor worker (including peasant, herdsman, fisherman, factory worker, and other labor worker) and office worker (including professional, manager, clerk and service worker). Education was classified as none, less than high school education, and some high school education or higher. Cigarette smokers were defined as having smoked at least one cigarette per day for one or more years. Alcohol consumption was defined as drinking alcohol at least 12 times during the last year. Body weight and height were measured in light indoor clothing without shoes, using a standard protocol. Body mass index (BMI) was then calculated as weight in kilograms divided by height in meters squared. Participants were classified as underweight (BMI <18.5 kg/m^2^), normal weight (BMI 18.5-25 kg/m^2^), or overweight and obese (BMI ≥25 kg/m^2^)[17].

### Follow-up data collection

The follow-up study was conducted between 1999 and 2000 to obtain information on vital status and incidence of selected diseases (vascular disease and cancer). The follow-up study included tracking study participants or their proxies to a current address, performing in-depth interviews to ascertain disease status and vital information, and obtaining hospital records and death certificates. Of all deaths reported, vital information was provided by family members (75.0%), primary-care physicians (12.6%), other healthcare providers (3.8%), and others (employers, relatives or friends, 8.5%). If death occurred while a participant was hospitalized, the participant's hospital record, including medical history, findings from physical examination, laboratory findings, autopsy findings, and discharge diagnosis, was abstracted by trained staff using a standard form. If death occurred outside of the hospital, a detailed medical history was obtained from a family member or healthcare provider. Participants' previous medical records were also obtained if available. The majority of deaths (98.6%) reported during follow-up were verified by death certificates and/or medical records.

A study-wide endpoint assessment committee reviewed medical history information and death certificates and determined the final underlying cause of death. Two committee members independently verified the diagnosis, and discrepancies were adjudicated by discussion involving additional committee members. All committee members were blinded to the study participant's baseline risk factor information. For the present analysis, there was perfect agreement between cause of death as recorded in the hospital records and death certificates. Causes of death were coded according to the International Classification of Diseases, Ninth Revision (ICD-9). Suicide and self-inflicted injury were coded as E950-E959. Other external cause injuries included transport accidents (E800-E848), accidental poisoning (E850-E869), misadventures to patients during surgical and medical care (E870-E879), accidental falls (E880-E888), accidents caused by fire and flames (E890-E899), accidents due to natural and environmental factors and accidents due to being struck by a falling object (E900-E909, E916), accidental drowning (E910), and homicide and injuries due to legal intervention (E960-E978).

The current study was approved by the Tulane University Health Sciences Center Institutional Review Board and the Cardiovascular Institute and Fu Wai Hospital Ethics Committee. Written documentation of informed consent was obtained from all study participants at their follow-up visit.

### Statistical analysis

Person-years were calculated from the baseline until death or follow-up interview. Deaths from suicide and other external cause injuries were grouped according to sex and 5-year age categories. Age-standardized mortality was calculated using the 5-year age-specific mortality and age distribution from 2000 census data for China. Age-standardized cause-specific mortality was calculated separately for the following categories: gender, urban vs. rural residence, northern vs. southern residence, BMI categories, employment status, education level, and drinking and smoking habits. Cox proportional hazards regression models were used to estimate the relative risk of death by risk factors. Age- and multivariable-adjusted relative risks (RR) with 95% confidence intervals (CI) and 2-sided *P *values were calculated. In multivariable models, age, gender, geographic region (north vs. south), and urbanization (urban vs. rural) were adjusted. Missing data were imputed using linear regression for body mass index, education level, and smoking and drinking status. There was no significant difference when imputed data were included or excluded.

## Results

During a mean follow-up of 8.3 years (1,239,191 person-years), a total of 788 external cause deaths were documented (197 deaths from suicide, and 591 deaths from other external cause injuries) among the 158,666 study participants. Age-standardized mortality from overall external cause injuries, suicide, and other external cause injuries are presented in Table 1. The age-standardized mortality of overall external cause injuries was 58.7 per 100,000 (14.1 for suicide and 44.6 for other external cause injuries). The age-standardized mortality from suicide was similar in men and women while mortality from other external cause injuries was higher in men than in women. With increasing age, mortality from suicide and other external cause injuries significantly increased. Underweight people (BMI <18.5 kg/m^2^) had the highest mortality and overweight people (BMI ≥25 kg/m^2^) had the lowest mortality from suicide and other external cause injuries. Mortality from other external causes was higher among individuals who lived in south China than those who lived in north China while mortality from suicide was similar in north and south China. Rural residents had higher mortality from suicide and other external cause injuries than urban residents. With respect to employment status, laborers and unemployed persons had higher mortality from suicide than office workers. Individuals without any schooling had highest mortality and those who went to high school or attained a higher education level had lowest mortality from suicide and other external cause injuries. Individuals who smoked or consumed alcohol had higher mortality from other external cause injuries than those who did not.

**Table 1:** Age-Standardized Mortality from Overall External Cause Injuries, Suicide and Other External Cause Injuries, China, 1991-1999

Sociodemographic characteristics	Overall external cause injuries			Suicide			Other external cause injuries		
	
	No. of death	Age-standardized rate/100 000 person-year	*P *value (trend)	No. of death	Age-standardized rate/100 000 person-year	*P *value (trend)	No. of death	Age-standardized rate/100 000 person-year	*P *value (trend)
Total	788	58.7		197	14.1		591	44.6	
Sex									
Female	306	44.4		99	14.2		207	30.2	
Male	482	72.3	<0.0001	98	14.2	0.66	384	58.1	<0.0001
Age (years)									
40-49	196	27.2		56	7.2		140	20.0	
50-59	175	32.1		49	9.4		126	22.6	
60-69	216	55.4		46	11.7		170	43.7	
≥ 70	201	266.3	<0.0001	46	32.6	<0.0001	155	233.7	<0.0001
BMI category (kg/m^2^)									
≥ 25	82	30.7		20	6.6		62	24.1	
18.5-24.9	485	63.1		120	14.7		365	48.4	
<18.5	137	96.1	<0.0001	32	23.5	<0.0001	105	72.5	<0.0001
Region									
South	357	61.5		82	14.6		275	46.9	
North	431	56.7	0.03	115	13.7	0.96	316	43.0	0.02
Urbanization									
Urban	224	31.1		45	5.4		179	25.7	
Rural	564	94.9	<0.0001	152	25.3	<0.0001	412	69.6	<0.0001
Employment status									
Office worker	49	41.6		8	3.6		41	37.9	
Unemployed	273	73.1		65	20.9		208	52.2	
Labor worker^†^	317	81.0	0.08	84	19.7	0.02	233	61.3	0.53
Education									
At least some HS	61	25.6		12	3.9		49	21.7	
Less than HS	272	61.3		60	12.4		212	48.9	
None	374	73.3	<0.0001	100	20.9	<0.0001	274	52.4	<0.0001
Smoking									
Never	379	48.0		103	12.8		276	35.2	
Ever smoker	326	75.6	<0.0001	67	14.3	0.57	259	61.3	<0.0001
Drinking									
Never	528	53.3		136	13.6		392	39.7	
Drinker	177	80.6	<0.0001	34	12.7	0.79	143	67.9	<0.0001

Abbreviations: BMI = body mass index; HS = high school

^† ^Labor worker includes peasant, herdsman, fisherman, factory worker, and other labor worker; office worker includes professional, manager, clerk and service worker.

**Figure 1:**
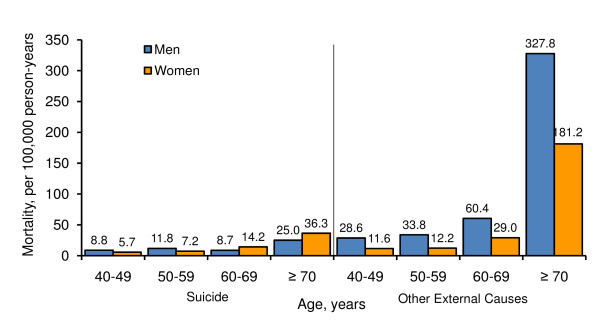
**Age-Specific Mortality Rates of Suicide and Other External Cause Injuries by Gender, China, 1991-1999**.

The age-specific mortality rates for suicide and other external cause injuries by gender are shown in Figure 1. For suicide, men had higher mortality than women before 60 years of age; however, the mortality reversed after 60 years of age. For other external cause injuries, men had higher mortality than women consistently across all age groups. In addition, mortality increased dramatically after 60 years of age for both suicide and other external cause injuries.

**Figure 2:**
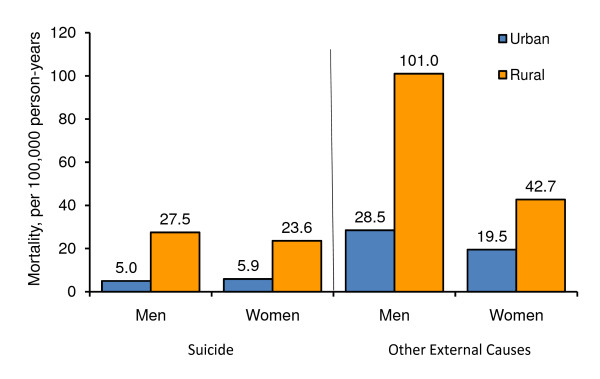
**Mortality Rates of Suicide and Other External Cause Injuries According to Gender and Urbanization in China, 1991-1999**.

The mortality rates from suicide and other external cause injuries by gender are shown in Figure 2 according to urbanization (urban vs. rural). Rural areas had higher mortality than urban areas for both suicide and other external cause injuries. For suicide, mortality of rural men was 5.5 times higher than that of urban men while mortality of rural women was 4 times higher than that of urban women. For other external cause injuries, mortality of rural men was 3.5 times higher than that of urban men while mortality of rural women was 2.2 times higher than that of urban women.

**Figure 3:**
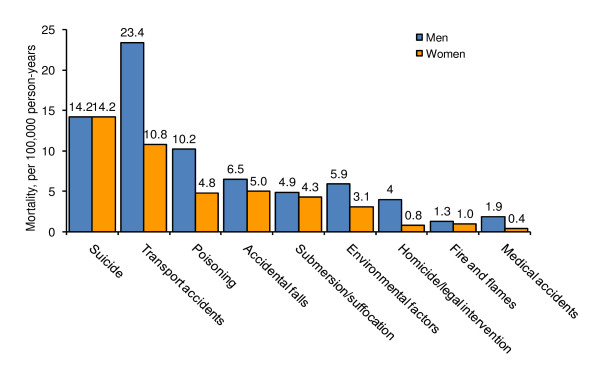
**Distribution of Causes of External Injuries, China, 1991-1999**.

The major causes of mortality from external injuries are shown in Figure 3. While suicide mortality rates are equal for men and women, mortality due to each of the other external cause injuries is consistently higher among men than women. The highest other external cause mortality rate is due to transport accidents followed by accidental poisoning, accidental falls, accidental drowning, accidents due to natural or environmental causes, homicide and legal intervention, accidents caused by fire and flames, and from misadventures to patients during surgical and medical care. The percentage of total external causes deaths were 28.8% from transport accidents, 25.0% from suicides, 13.1% from accidental poisoning, 10.7% from accidental falls, 7.6% from accidental drowning, 7.5% from accidents due to natural and environmental factors, 3.4% from homicide and legal intervention, 2.2% from accidents caused by fire and flames, and 1.8% from misadventures to patients during surgical and medical care.

Age- and multivariable-adjusted RRs (95% CI) for death due to suicide and other external cause injuries are presented in Table 2. For example, in the multivariable-adjusted models, older age, living in north China, and rural residence were associated with increased risk of mortality from suicide. Male sex, older age, rural residence, being underweight, and having less than a high school education or no education were associated with increased risk of mortality from other external cause injuries, while being overweight or obese was associated with decreased risk of mortality from other external cause injuries in multivariable-adjusted models.

**Table 2:** Age Adjusted and Multivariable Adjusted Relative Risk According to Suicide and Other External Cause Injuries, China, 1991-1999

Sociodemographic characteristics	Suicide (n = 197)						Other external cause injuries (n = 591)					
	
	Age-adjusted			Multivariable-adjusted^*^			Age-adjusted			Multivariable-adjusted^*^		
	
	RR	95% CI	p-value	RR	95% CI	p-value	RR	95% CI	p-value	RR	95% CI	p-value
Sex												
Female	1.00	reference		1.00	reference		1.00	reference		1.00	reference	
Male	1.03	0.78-1.36	0.83	1.15	0.87-1.52	0.34	1.93	1.63-2.29	<0.0001	2.19	1.85-2.60	<0.0001
Age (years)												
40-49	1.00	reference		1.00	reference		1.00	reference		1.00	reference	
50-59	1.01	0.69-1.49	0.95	1.09	0.74-1.60	0.66	1.04	0.82-1.33	0.73	1.11	0.87-1.42	0.39
60-69	1.46	0.99-2.16	0.06	1.56	1.05-2.31	0.03	2.17	1.74-2.72	<0.0001	2.28	1.82-2.86	<0.0001
≥ 70	3.34	2.26-4.94	<0.0001	3.60	2.43-5.34	<0.0001	4.55	3.62-5.72	<0.0001	4.84	3.84-6.09	<0.0001
Region												
South	1.00	reference		1.00	reference		1.00	reference		1.00	reference	
North	0.97	0.73-1.29	0.84	1.41	1.05-1.89	0.02	0.78	0.67-0.92	<0.01	1.06	0.89-1.25	0.53
Urbanization												
Urban	1.00	reference		1.00	reference		1.00	reference		1.00	reference	
Rural	4.43	3.18-6.18	<0.0001	3.97	2.72-5.79	<0.0001	3.04	2.55-3.62	<0.0001	3.02	2.47-3.70	<0.0001
BMI category (kg/m^2^)												
< 18.5	1.59	1.07-2.34	0.02	1.22	0.81-1.82	0.34	1.72	1.38-2.13	<0.0001	1.14	0.91-1.43	0.26
18.5-24.9	1.00	reference		1.00	reference		1.00	reference		1.00	reference	
≥ 25	0.46	0.28-0.73	<0.01	0.66	0.40-1.08	0.10	0.46	0.35-0.61	<0.0001	0.69	0.52-0.92	0.01
Employment status												
Office worker	1.00	reference		1.00	reference		1.00	reference		1.00	reference	
Labor worker*	4.72	2.28-9.74	<0.0001	1.41	0.62-3.22	0.41	2.56	1.84-3.56	<0.0001	1.20	0.81-1.76	0.37
Unemployed	5.33	2.56-11.1	<0.0001	1.93	0.85-4.41	0.12	3.34	2.39-4.67	<0.0001	1.32	0.89-1.95	0.16
Education												
At least some HS	1.00	reference		1.00	reference		1.00	reference		1.00	reference	
Less than HS	2.87	1.55-5.34	<0.01	1.55	0.80-3.01	0.20	2.48	1.82-3.38	<0.0001	1.54	1.10-2.14	0.01
None	5.90	3.24-10.8	<0.0001	2.00	0.98-4.06	0.06	3.96	2.92-5.37	<0.0001	1.79	1.24-2.57	<0.01
Smoking												
Never	1.00	reference		1.00	reference		1.00	reference		1.00	reference	
Ever smoker	1.07	0.78-1.45	0.69	0.93	0.64-1.35	0.71	1.54	1.30-1.82	<0.0001	1.09	0.89-1.33	0.41
Drinking												
Never	1.00	reference		1.00	reference		1.00	reference		1.00	reference	
Drinker	1.00	0.69-1.45	0.99	0.91	0.59-1.38	0.64	1.46	1.20-1.77	<0.0001	1.11	0.90-1.38	0.32

RR = relative risk; CI = confidence interval; BMI = body mass index; HS = high school

* Multivariable adjusted RR was calculated by adjusting for age, sex, region (north vs. south), and urbanization (urban vs. rural). Models included stratification by province to adjust for cluster sampling, except for the model for which region (north vs. south) was the main study variable.

^† ^Labor worker includes peasant, herdsman, fisherman, factory worker, and other labor worker; office worker includes professional, manager, clerk and service worker

In the multivariable model which included all variables of interest simultaneously (Table 3), male sex, older age, living in north China, and rural residence increased the risk of mortality from suicide. Male sex, older age, rural residence, and having no education were associated with increased risk of mortality from other external cause injuries, whereas overweight or obese status was associated with decreased risk of other external cause injury mortality.

**Table 3:** Multivariable Adjusted Relative Risk of Suicide and Other External Cause Injuries in China, 1991-1999*

Sociodemographic characteristics	Suicide (n = 197)			Other external cause (n = 591)		
	
	Multivariable adjusted			Multivariable adjusted		
	
	RR	95% CI	p-value	RR	95% CI	p-value
Sex						
Female	1.00	reference		1.00	reference	
Male	1.56	1.03-2.38	0.04	2.50	1.95-3.20	<0.0001
Age (years)						
40-49	1.00	reference		1.00	reference	
50-59	0.90	0.58-1.41	0.65	1.11	0.85-1.45	0.47
60-69	1.11	0.67-1.84	0.69	1.93	1.45-2.58	<0.0001
≥ 70	2.27	1.29-3.98	<0.01	3.58	2.58-4.97	<0.0001
Region						
South	1.00	reference		1.00	reference	
North	1.68	1.20-2.36	<0.01	1.09	0.90-1.31	0.38
Urbanization						
Urban	1.00	reference		1.00	reference	
Rural	2.82	1.76-4.51	<0.0001	2.29	1.77-2.96	<0.0001
BMI category (kg/m^2^)						
< 18.5	1.19	0.79-1.79	0.42	1.10	0.87-1.39	0.41
18.5-24.9	1.00	reference		1.00	reference	
≥ 25	0.61	0.35-1.08	0.09	0.60	0.43-0.83	<0.01
Employment status						
Office worker	1.00	reference		1.00	reference	
Labor worker^†^	1.33	0.54-3.26	0.54	0.96	0.63-1.48	0.87
Unemployed	1.70	0.68-4.21	0.26	1.25	0.81-1.91	0.32
Education						
At least some HS	1.00	reference		1.00	reference	
Less than HS	1.40	0.59-3.32	0.45	1.34	0.89-2.02	0.16
None	1.92	0.78-4.76	0.16	1.56	1.00-2.43	0.05
Smoking						
Never	1.00	reference		1.00	reference	
Ever smoker	0.81	0.55-1.21	0.31	1.02	0.82-1.27	0.84
Drinking						
Never	1.00	reference		1.00	reference	
Drinker	1.11	0.72-1.71	0.65	1.05	0.84-1.32	0.67

RR = relative risk; CI = confidence interval; BMI = body mass index; HS = high school

* All variables for which values are listed are included in the model.

^† ^Labor worker includes peasant, herdsman, fisherman, factory worker, and other labor worker; office worker includes professional, manager, clerk and service worker.

## Discussion

The current study documents death due to external causes as a major public health challenge in China, reporting an overall mortality rate of 58.7 deaths per 100,000 Chinese residents annually. These estimates include 14.1 and 44.6 deaths (per 100,000) due to suicide and other external cause injuries, respectively. Notably, and in contrast to previous findings, we identified male gender as an important predictor of increased suicide mortality. Other risk factors for suicide included older age and residency in rural and North China. Transport accidents, accidental poisoning, and accidental falls were the leading causes of other external cause injuries in China. Male gender, older age, rural residence, and having no education were associated with increased mortality from other external cause injuries, while overweight appeared to decrease mortality from other external cause injuries. The present results emphasize the importance of death from external cause injury and highlight the need for a public health response focusing on its prevention in the Chinese population.

Suicide is a major cause of premature death and thus is an important public health issue worldwide, particularly in China. In this study, we estimated suicide mortality to be 14.1 per 100,000, which is lower than previous reports potentially due to the exclusion of younger individuals in our study. Phillips' study reported an average suicide mortality rate of 23 per 100,000 yearly during the period of 1995-1999 in China[2]. Yip and colleagues document a decrease in suicide mortality from 22.9 in 1991 to 15.4 per 100,000 in 2000[9]. Qin and colleagues projected national suicide rates of 17.7-22.6 per 100,000 during the period of 1987-1994[5]. All of the previous studies used vital statistical data provided by the Chinese Ministry of Health[2,5,15]. Due to the lack of a national death registration system, the validity of these data could not be confirmed. To the best of our knowledge, the current study is the first large prospective study conducted in China to estimate suicide mortality in a nationally representative adult population.

Our study showed that suicide was 2.8 times more common in rural areas than in urban areas. Similar findings have been shown in other studies in China and in other populations across the world[2,5,7,8,15,18]. Some factors associated with living in a rural environment that may contribute to an increased suicide rate include lower socioeconomic status, traditional belief system, and less developed social networks[5,19]. Easier access to highly poisonous agents such as pesticides and more difficult access to health services in rural China could lead to a higher proportion of completed suicide attempts[2,6].

Notably, we found a slight increase risk of suicide for men after adjusted for multiple co-variables. In our study, suicide mortality is higher for men in middle-age groups (<60 years) and higher for women in the older age groups (≥60 years). By urbanization, suicide mortality for rural men exceeds that of rural women, whereas this relationship is reversed in urban areas. The majority of the literature identifies China as being one of few countries with a higher suicide rate for women than men, which seems to be primarily attributable to an excess of suicide fatalities among younger women residing in rural areas[2,5-7,9]. Younger persons (<40 years), however, were excluded from our study. The findings of Parker and colleagues also show an increase in the male to female ratio for suicide in Singapore during the 1990s, with a relatively greater decrease for females, and especially among the Chinese[20].

The elderly are particularly susceptible to suicide[6,21]. In the present study, suicide rates dramatically increased with age. Suicide risk among the elderly could be a consequence of increased terminal illness, disability, and depression[6,22,23]. With regard to other suicide risk factors that have been suggested in prior studies [7,13,24,25], although we found significant associations between BMI, employment and education with suicide, these were not significant after adjustment. In addition, we document residence in north China as a risk factor and found that smoking and drinking habits were not related to suicide.

Transport injuries rank among the leading causes of death worldwide[1]. Among transport injuries, motor vehicle accidents are responsible for the majority of deaths, particularly in younger age groups[2]. In our study, transport accidents accounted for the majority (28.8%) of overall external cause fatalities. Accidental drowning, falls, and poisoning are also major causes of injury death, which is consistent with the World Health Organization Global Burden of Disease estimates[1]. It is possible that some suicides committed by ingesting poison such as pesticides were misclassified as accidental poisoning deaths, thereby overestimating the number of deaths due to accidental poisoning and underestimating the number of deaths due to suicide. However, mortality from all external causes should be accurate because both suicide deaths and other external cause injuries were examined in the current study.

The overall mortality due to other external cause injuries was 44.6 per 100 000 in the study population. Men had a higher rate of mortality due to other external cause injuries compared to women. Elderly persons are particularly vulnerable to death resulting from injury due to increased susceptibility to complications and given that there is poor access to medical care in rural China[26]. In addition to age and sex, other socio-demographic factors and health behaviors, such as urbanization and being uneducated, are related to injuries after accounting for covariates. Being overweight was associated with lower risk of death due to other external cause injuries in this study population.

The principal strength of our study is its prospective cohort design and large, national representative sample. Due to the lack of a complete death registry, accurate estimates of mortality from external cause injuries in China were not previously available. Given the sampling methods employed, these results can be generalized to the overall Chinese population and possibly to other countries with similar characteristics. Furthermore, accuracy of cause of death was ensured by death certificates and/or hospital records in our study. We examined the association of socio-demographic risk factors that were not investigated in previous studies, such as BMI category, employment status, education level, smoking and drinking on mortality from external cause injuries in China.

Due to the national representativeness of this study, the identification of demographic factors and other characteristics can be used to develop national programs for the prevention of external cause injury mortality. For the prevention of injuries and violence, the World Health Organization advises enforcement of seat-belt use and helmets, restricting access to means of committing suicide such as pesticides, limiting access to bodies of water, improving health services for victims of injury and violence and developing crisis centers[27,28]. In a systematic review of suicide prevention programs worldwide, physician education and reducing access to lethal means to commit suicide were found to prevent suicide[29]. Continued research to assess patterns in rates of suicide and other external cause injuries and to evaluate prevention programs is warranted.

## Conclusions

Suicide and other external cause injuries are major public health problems in China. National prevention programs should be established to reduce mortality burden from external cause injuries in China.

## Competing interests

The authors declare that they have no competing interests.

## Authors' contributions

DG and JH are principal investigators. DG, XW, JC, and JH designed the study. DG, XW, J-FH, J-CC, XD, and JH oversaw data collection. Analysis was undertaken by CMR, WY, and C-SC under supervision of JH. CMR, WY and JH drafted the paper. All authors contributed to the interpretation of the data and have approved the final version. JH is the guarantor for the study.

## Pre-publication history

The pre-publication history for this paper can be accessed here:

http://www.biomedcentral.com/1471-2458/11/56/prepub
